# Prediction of Acute Kidney Injury Following Isolated Coronary Artery Bypass Grafting in Heart Failure Patients with Preserved Ejection Fraction Using Machine Leaning with a Novel Nomogram

**DOI:** 10.31083/j.rcm2502043

**Published:** 2024-01-29

**Authors:** Xuejian Hou, Kui Zhang, Taoshuai Liu, Shijun Xu, Jubing Zheng, Yang Li, Ran Dong

**Affiliations:** ^1^Department of Cardiac Surgery, Beijing Anzhen Hospital, Capital Medical University, 100000 Beijing, China

**Keywords:** heart failure with preserved ejection fraction (HFpEF), acute kidney injury (AKI), coronary artery bypass grafting (CABG), machine learning

## Abstract

**Background:**

The incidence of postoperative acute kidney injury (AKI) is high due to insufficient perfusion in patients with heart failure. Heart failure patients with preserved ejection fraction (HFpEF) have strong heterogeneity, which can obtain more accurate results. There are few studies for predicting AKI after coronary artery bypass grafting (CABG) in HFpEF patients especially using machine learning methodology.

**Methods:**

Patients were recruited in this study from 2018 to 2022. AKI was defined according to the Kidney Disease Improving Global Outcomes (KDIGO) criteria. The machine learning methods adopted included logistic regression, random forest (RF), extreme gradient boosting (XGBoost), gaussian naive bayes (GNB), and light gradient boosting machine (LGBM). We used the receiver operating characteristic curve (ROC) to evaluate the performance of these models. The integrated discrimination improvement (IDI) and net reclassification improvement (NRI) were utilized to compare the prediction model.

**Results:**

In our study, 417 (23.6%) patients developed AKI. Among the five models, random forest was the best predictor of AKI. The area under curve (AUC) value was 0.834 (95% confidence interval (CI) 0.80–0.86). The IDI and NRI was also better than the other models. Ejection fraction (EF), estimated glomerular filtration rate (eGFR), age, albumin (Alb), uric acid (UA), lactate dehydrogenase (LDH) were also significant risk factors in the random forest model.

**Conclusions:**

EF, eGFR, age, Alb, UA, LDH are independent risk factors for AKI in HFpEF patients after CABG using the random forest model. EF, eGFR, and Alb positively correlated with age; UA and LDH had a negative correlation. The application of machine learning can better predict the occurrence of AKI after CABG and may help to improve the prognosis of HFpEF patients.

## 1. Introduction

The incidence of acute kidney injury (AKI) after coronary artery bypass grafting (CABG) is high and has been reported to range from 6.7% to 39% [1, 2, 3]. AKI has been associated with increased morbidity and mortality after CABG [3, 4, 5], which further increases in the more severe stages of AKI; and is associated with increased short-term and long-term mortality [4, 6, 7, 8, 9]. AKI after cardiac surgery also increases intensive care unit (ICU) length of stay and resource utilization [2, 10].

The development of AKI involves a variety of mechanisms, including ischemic reperfusion injury, renal toxin release, hemolysis, oxidative stress and cytokine secretion, which can cause a systemic inflammatory response, endothelial damage and renal tubular cell damage [1, 11, 12, 13]. Previous studies have shown that older age, low ejection fraction, a previous history of kidney disease, and increased time on cardiopulmonary bypass are important predictors of the development of AKI [6, 14, 15, 16].

While patients with heart failure and reduced ejection fraction are more likely to develop AKI after CABG, patients with preserved ejection fraction may also develop AKI after surgery which will also seriously affect the prognosis of those patients. The research methods for disease risk prediction models are constantly being updated. The introduction of machine learning methods now offers another technique to predict the occurrence of adverse events following surgery [17]. Traditional logistic analysis generally deals with data with appropriate size, single types of data, structured data and simple parameter models that meet certain assumptions. For large and complex data, machine learning methods can be used to obtain more accurate risk prediction. In this study, we sought to predict AKI in patients with preserved ejection fraction after isolated CABG using machine learning methodology.

## 2. Methods

### 2.1 Patients and Setting 

1767 patients who underwent CABG for the first time from 2018 to 2022 were recruited in this study. According to the Kidney Disease Improving Global Outcomes (KDIGO) diagnostic criteria of AKI [18], patients divided into developed AKI (AKI group) and who did not (non-AKI group).

### 2.2 Definition of AKI 

AKI was defined according to the KDIGO criteria [18]: an increase in serum creatinine (Scr ≥0.3 mg/dL) or an increase in Scr ≥1.5 times baseline in 7 days after surgery or urine volume ≤0.5 mL/kg/h for 6 h.

### 2.3 Definition of Heart Failure with Preserved Ejection Fraction (HFpEF) 

(1) Symptoms and signs of heart failure (HF); (2) An left ventricular ejection fraction (LVEF) ≥50%; (3) Objective evidence of cardiac structural and/or functional abnormalities consistent with the presence of left ventricle (LV) diastolic dysfunction/raised LV filling pressures, including raised brain natriuretic peptide (BNP) (>35 (sinus rhythm)) or >105 (atrial fibrillation) pg/mL [19].

### 2.4 Data Collection

Detailed clinical information included age, sex, body mass index (BMI), previous cardiac history (previous myocardial infarction and previous percutaneous coronary intervention (PCI)), diabetes, hypertension, Carotid-artery-stenosis, previous stroke or chronic obstructive pulmonary disease, smoking, baseline renal function (eGFR, estimated glomerular filtration rate), anemia, and preoperative intra-aortic balloon pump (IABP) implantation.

### 2.5 Model Development

**Fig. 1.**
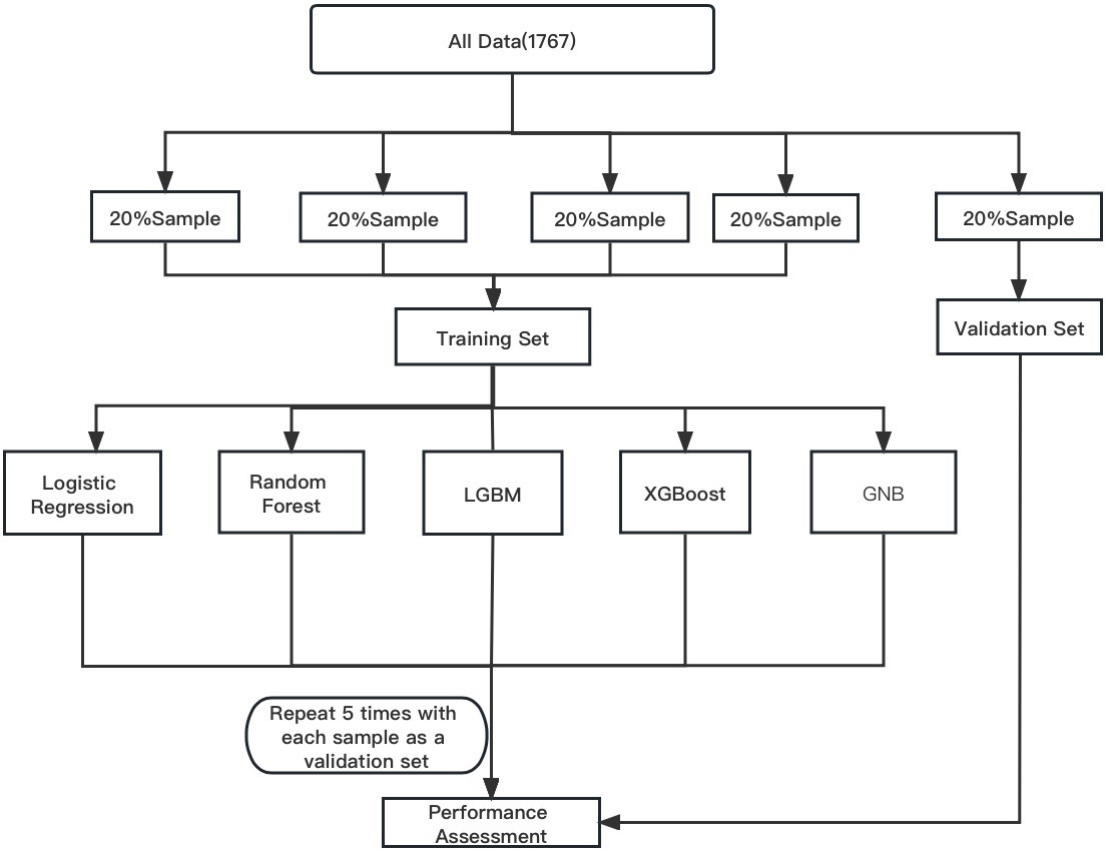
**Analysis flow for the development and evaluation of models**. LGBM, light gradient boosting machine; XGBoost, extreme gradient boosting; GNB, gaussian naive bayes.

We used logistic regression, random forest (RF), extreme gradient boosting (XGBoost), gaussian naive bayes (GNB), light gradient boosting machine (LGBM), and logistic regression (LR) algorithms to filter out significant variables. The significant variables are derived to train and verify the model. In our study, 80% of the population were merged to form a training group, while the remaining (20%) served as the verification group. The process was repeated five times for each result so that each subset can be used for a validation set to explain the differences between patients and provide risk estimates for all cases. The software (version: 4.1.0) packages including XGB Classifier, LGBM Classifier, sklearn naive bayes, sklearn model selection, sklearn.metrics, sklearn.ensemble were used for analysis as shown in Fig. 1. We used the receiver operating characteristic curve (ROC) to evaluate the performance of these models. The integrated discrimination improvement (IDI) and net reclassification improvement (NRI) also were used to evaluate the prediction model.

### 2.6 Outcome Measures 

The most important variables were screened out by the five models, and then the area under curve (AUC), NRI and IDI of each model were compared. By comparing AUC values, the best prediction model was selected. Then the calibration of the best model was checked. The most significant factors are included in the nomogram.

### 2.7 Statistical Analysis

SPSS 23.0 for Mac (IBM SPSS Statistics, Armonk, NY, USA), R (version 4.1.0, Lucent Technologies, Murray Hill, NJ, USA) and Python (version 3.5) were used for statistical analysis. Continuous variables were reported as the mean standard deviation or median (interquartile range (IQR)). Categorical variables were reported as the absolute frequency and as a percentage. Student’s *t*-test was applied for continuous data with equal or unequal variances. The Mann-Whitney U test was applied for continuous data that were not normally distributed. Pearson’s χ^2^ and Fisher’s exact tests were used for categorical data. A *p* < 0.05 was considered to be statistically significant.

## 3. Results

### 3.1 Patient Characteristics

**Fig. 2.**
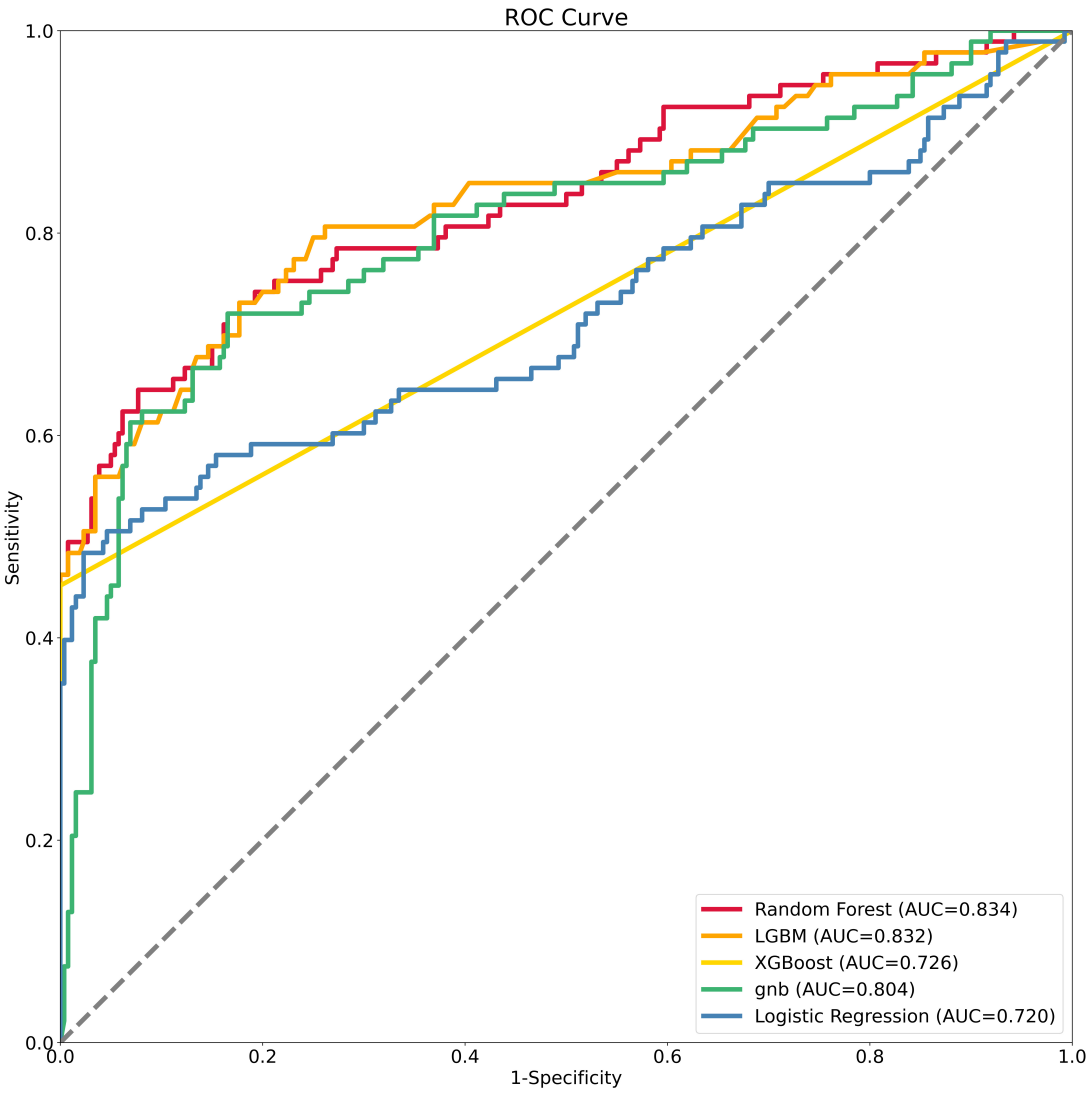
**The comparison of receiver operating characteristic curve (ROC) curves among the five models**. LGBM, light gradient boosting machine; XGBoost, extreme gradient boosting; gnb, gaussian naive bayes; AUC, area under curve.

1767 patients with HFpEF were included. The baseline clinical data train and test groups are shown in Table 1. There was no significant difference in baseline statistical results between the training group and the verification group. The incidence of AKI was 23.6%. The comparison of ROC curves among the five models is shown in Fig. 2. The RF performed best with the highest C-statistic (0.834 95% CI 0.80–0.86, Brier score: 0.142, NRI: 0.044, IDI: 0.172). The results if the other models are shown in Table 2.

**Table 1.** **Characteristics of patients in training and testing group**.

		Overall	Test	Train	*p*
		1767	353	1414	
BNP (pg/L) (median (IQR))		280 (179, 446)	294 (183, 488)	275 (177, 473)	0.21
EF (%) (median (IQR))		58 (54, 60)	58 (54, 60)	58 (54, 60)	0.92
ICU stay (h) (median (IQR))		21.3 (16.8, 39.9)	21.4 (17.8, 40.2)	21.2 (16.6, 39.7)	0.19
Hb (g/L)		112.65 (22.69)	113.57 (24.10)	112.42 (22.33)	0.40
ALT (U/L)		34.31 (61.14)	39.67 (73.61)	32.97 (57.56)	0.07
AST (U/L)		33.31 (44.07)	37.31 (45.09)	32.31 (43.77)	0.06
TG (mmol/L)		1.60 (0.92)	1.63 (0.90)	1.59 (0.92)	0.50
TC (mmol/L)		3.92 (1.04)	3.84 (0.92)	3.95 (1.07)	0.08
HDL (mmol/L)		0.98 (0.23)	0.98 (0.24)	0.98 (0.23)	0.98
LDL (mmol/L)		2.31 (0.88)	2.22 (0.77)	2.34 (0.91)	0.03
Urea (mmol/L)		6.67 (2.67)	6.62 (2.62)	6.68 (2.68)	0.71
Cr (umol/L)		73.61 (21.24)	74.33 (19.88)	73.43 (21.58)	0.48
eGFR (umol/L)		90.12 (17.92)	90.07 (16.91)	90.14 (18.16)	0.95
UA (umol/L)		333.01 (91.22)	330.60 (94.81)	333.61 (90.32)	0.58
CK (U/L)		8.54 (21.14)	9.83 (20.77)	8.22 (21.23)	0.20
TP (g/L)		67.80 (5.73)	68.15 (5.55)	67.72 (5.77)	0.21
Alb (g/L)		41.50 (3.61)	41.69 (3.52)	41.45 (3.64)	0.26
TB (umol/L)		12.12 (5.86)	12.39 (5.81)	12.05 (5.87)	0.33
DB (umol/L)		3.90 (2.03)	3.93 (1.88)	3.90 (2.07)	0.77
WBC (${\mathrm{10}}^{9}$/L)		10.19 (4.13)	9.96 (3.76)	10.25 (4.22)	0.23
RBC (${\mathrm{10}}^{12}$/L)		3.67 (0.74)	3.70 (0.79)	3.66 (0.73)	0.45
PLT (${\mathrm{10}}^{9}$/L)		205.71 (76.72)	203.72 (79.06)	206.20 (76.15)	0.59
NE (%)		76.13 (11.82)	75.76 (12.00)	76.22 (11.78)	0.51
HCT (%)		32.83 (6.48)	33.03 (6.88)	32.78 (6.38)	0.52
Glu (mmol/L)		8.27 (3.35)	8.54 (3.59)	8.20 (3.29)	0.09
Hcy (ummol/L)		16.83 (9.46)	16.79 (9.09)	16.84 (9.56)	0.93
LDH (U/L)		203.72 (101.27)	205.65 (98.15)	203.24 (102.06)	0.69
Mb (ng/mL)		244.19 (286.28)	239.20 (243.01)	245.44 (296.17)	0.71
Ca (mmol/L)		2.17 (0.20)	2.16 (0.21)	2.17 (0.20)	0.25
ALP (U/L)		80.40 (27.86)	81.85 (32.18)	80.03 (26.67)	0.27
GGT (U/L)		37.04 (37.01)	37.27 (40.89)	36.98 (36.00)	0.89
Age (median (IQR))		64 (58, 69)	64 (57, 68)	65 (58, 69)	0.08
Sex (%)					
	Female	458 (25.9)	89 (25.2)	369 (26.1)	0.74
	Male	1309 (74.1)	264 (74.8)	1045 (73.9)	
Hight (cm)		166.44 (7.82)	166.76 (7.80)	166.36 (7.83)	0.39
Weight (kg)		71.58 (11.39)	71.30 (11.49)	71.64 (11.36)	0.61
BMI		25.77 (3.25)	25.56 (3.17)	25.82 (3.27)	0.17
Inhospital days (d)		14.52 (5.80)	14.55 (5.81)	14.51 (5.80)	0.90
On-pump (%)					
	Yes	1391 (78.7)	282 (79.9)	1109 (78.4)	0.55
	No	376 (21.3)	71 (20.1)	305 (21.6)	
Re-thoracotomy (%)					
	No	1734 (98.1)	346 (98.0)	1388 (98.2)	0.86
	Yes	33 (1.9)	7 (2.0)	26 (1.8)	
Preoperative IABP (%)					
	No	1680 (95.1)	330 (93.5)	1350 (95.5)	0.12
	Yes	87 (4.9)	23 (6.5)	64 (4.5)	
Ventilation >24 h (%)					
	No	1297 (73.4)	250 (70.8)	1047 (74.0)	0.22
	Yes	470 (26.6)	103 (29.2)	367 (26.0)	
Prior-MI (%)					
	No	1464 (82.9)	301 (85.3)	1163 (82.2)	0.18
	Yes	303 (17.1)	52 (14.7)	251 (17.8)	
Angina (%)					
	No	154 (8.7)	32 (9.1)	122 (8.6)	0.80
	Yes	1613 (91.3)	321 (90.9)	1292 (91.4)	
STEMI (%)					
	No	1695 (95.9)	337 (95.5)	1358 (96.0)	0.63
	Yes	72 (4.1)	16 (4.5)	56 (4.0)	
NSTEMI (%)					
	No	1685 (95.4)	337 (95.5)	1348 (95.3)	0.91
	Yes	82 (4.6)	16 (4.5)	66 (4.7)	
Hypertension (%)					
	No	615 (34.8)	123 (34.8)	492 (34.8)	0.99
	Yes	1152 (65.2)	230 (65.2)	922 (65.2)	
Diabetes (%)					
	No	1059 (59.9)	203 (57.5)	856 (60.5)	0.30
	Yes	708 (40.1)	150 (42.5)	558 (39.5)	
Previous stroke (%)					
	No	1549 (87.7)	294 (83.3)	1255 (88.8)	0.01
	Yes	218 (12.3)	59 (16.7)	159 (11.2)	
COPD (%)					
	No	1720 (97.3)	340 (96.3)	1380 (97.6)	0.18
	Yes	47 (2.7)	13 (3.7)	34 (2.4)	
Prior-PCI (%)					
	No	1581 (89.5)	324 (91.8)	1257 (88.9)	0.11
	Yes	186 (10.5)	29 (8.2)	157 (11.1)	
Carotid-artery-stenosis (%)					
	No	1698 (96.1)	341 (96.6)	1357 (96.0)	0.58
	Yes	69 (3.9)	12 (3.4)	57 (4.0)	
AF (%)					
	No	1723 (97.5)	342 (96.9)	1381 (97.7)	0.40
	Yes	44 (2.5)	11 (3.1)	33 (2.3)	
Family-history (%)					
	No	1711 (96.8)	345 (97.7)	1366 (96.6)	0.28
	Yes	56 (3.2)	8 (2.3)	48 (3.4)	
Smoking (%)					
	No	961 (54.4)	198 (56.1)	763 (54.0)	0.47
	Yes	806 (45.6)	155 (43.9)	651 (46.0)	
Drink (%)					
	No	1309 (74.1)	266 (75.4)	1043 (73.8)	0.54
	Yes	458 (25.9)	87 (24.6)	371 (26.2)	
EuroScore (median (IQR))		5 (4, 7)	5 (4, 6)	5 (4, 7)	0.65

IQR, interquartile range; eGFR, estimated glomerular filtration rate; Alb, albumin; LDH, lactate dehydrogenase; BMI, body mass index; IABP, intra-aortic balloon pump; PCI, percutaneous coronary intervention; EF, ejection fraction; ALT, alanine amiotransferase; AST, aspartate aminotransferase; TG, triglyceride; TC, total cholesterol; HDL, high density lipoprotein cholesterol; LDL, low density lipoprotein cholesterol; UA, uric acid; CK, creatine kinase; TP, total protein; TB, total bilirubin; DB, direct bilirubin; ALP, alkaline phosphatase; GGT, γ-glutamyl transpeptadase; WBC, white blood cell; RBC, red blood cell; PLT, platelet count; NE, neutrofili; HCT, hematocrit; STEMI, ST segment elevation myocardial infarction: NSTEMI, non-ST segment elevation myocardial infarcion; COPD, chronic obstructive pulmonary disease; AF, atrial fibrillation; MI, myocardial infarct; BNP, brain natriuretic peptide; ICU, intensive care unit; Hb, hemoglobin; Glu, glucose; Hcy, homocysteine; Mb, myoglobin.

**Table 2.** **Comparison of prediction effect evaluation of five models**.

Model	Precision	Recall	F1score	Accuracy	Brier	NRI	IDI
RF	0.462	1.000	0.632	0.858	0.142	0.044	0.172
LGBM	0.392	1.000	0.610	0.850	0.131	0.034	0.162
XGBoost	0.452	0.940	0.622	0.856	0.144	0.033	–0.185
GNB	0.430	0.769	0.552	0.816	0.184	–0.035	0.139
LR	0.430	0.930	0.588	0.841	0.159	0.000	0.000

Precision: Measure the precision of the model. Recall: It measures the recall of the retrieval system. F1score: It is the harmonic average of precision and recall. Brier: Evaluation of the overall performance of the model. IDI, integrated discrimination improvement; NRI, net reclassification improvement; RF, random forest; LR, logistic regression; LGBM, light gradient boosting machine; XGBoost, extreme gradient boosting; GNB, gaussian naive bayes.

### 3.2 Predictor Variables

The five models screened out the most important predictors. Random forest showed the best prediction effect. The ejection fraction (EF), eGFR, age, albumin (Alb), uric acid (UA), lactate dehydrogenase (LDH) were the most obvious risk factors in random forest.

### 3.3 Calibration of the Model

**Fig. 3.**
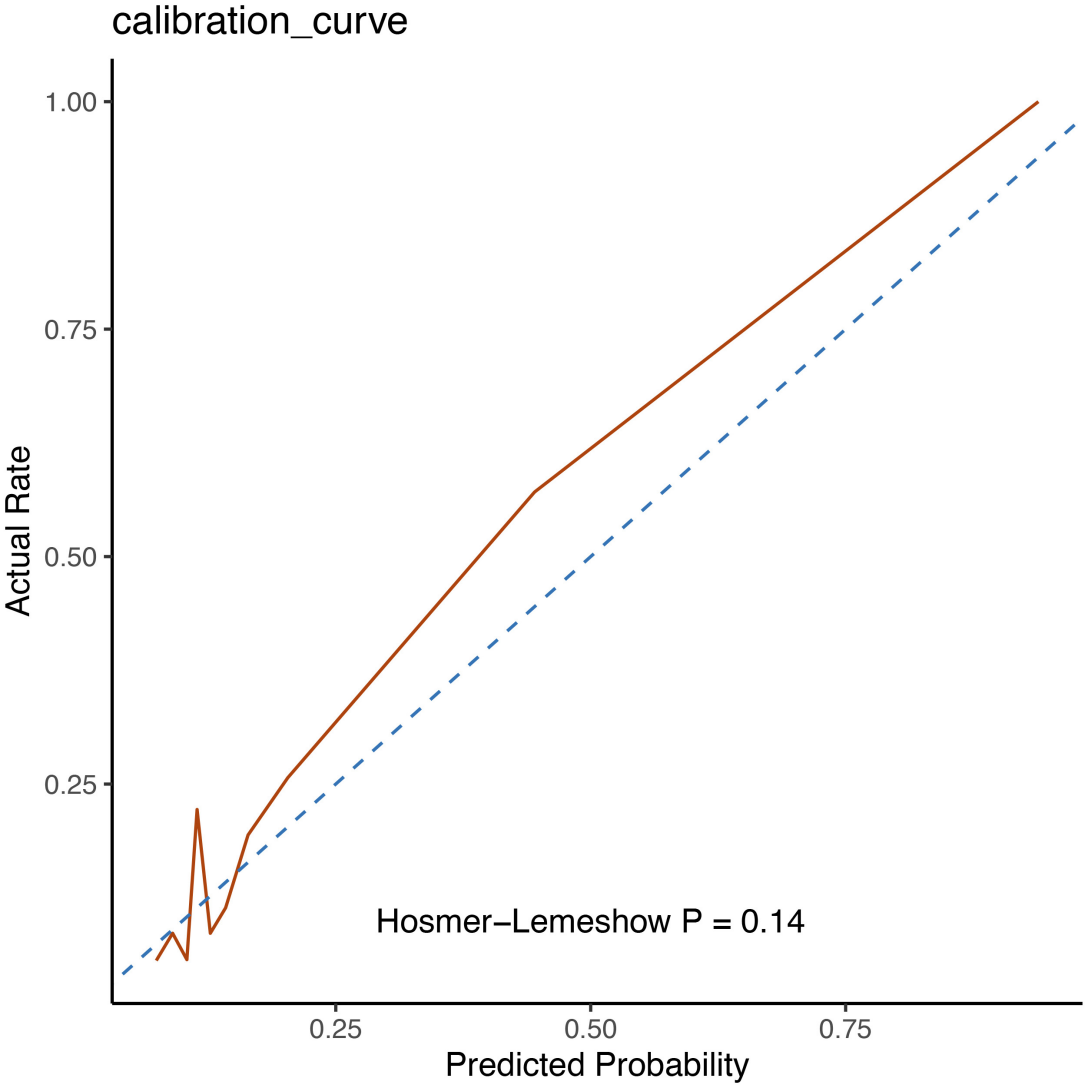
**The calibration of random forest model**.

The best model was calibrated. The Hosmer-Lemeshow good of fit test was used to evaluate the calibration degree of the predicted model. The *p *value was 0.14, which indicated that the calibration of the RF model was good. The calibration curve was shown in Fig. 3.

### 3.4 Construction of Tools for Patient Classification

**Fig. 4.**
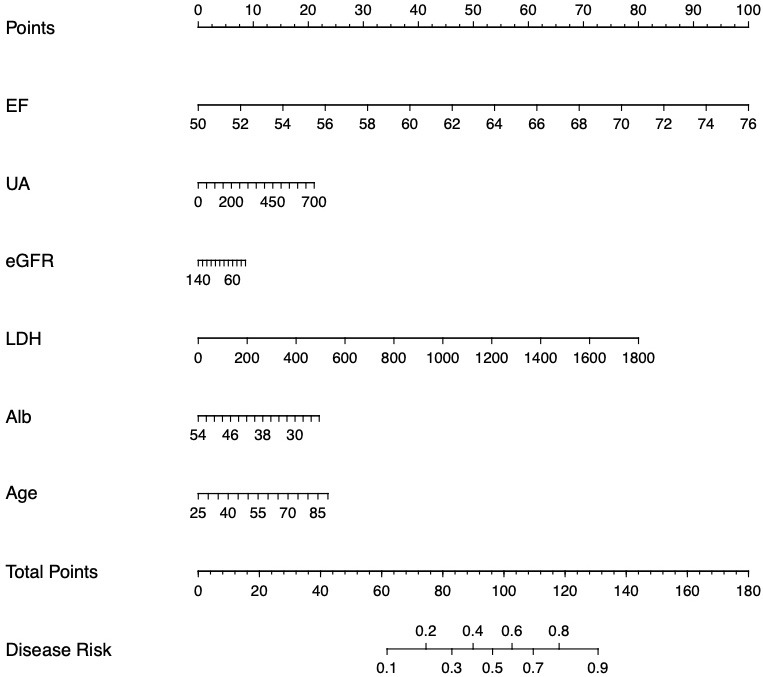
**The nomogram used to quantify the risk of AKI**. EF, ejection fraction; UA, uric acid; eGFR, estimated glomerular filtration rate; LDH, lactate dehydrogenase; Alb, albumin; AKI, acute kidney injury.

In order to calculate the probability of postoperative AKI, we included the most important risk factors in the nomogram. By using the nomogram we could quickly calculate the incidence of AKI and provide more accurate data for clinical practice. The nomogram was shown in Fig. 4.

## 4. Discussion

Our study used machine learning methods, whereas previous studies included a small population and mixed surgical types. Our results suggest that the EF, eGFR, age, Alb, UA, and LDH are independent risk factors for AKI in HFpEF patients after CABG using the random forest model. The incidence of AKI was 23.6% in our study which is similar to previous studies [1, 2, 3].

While there are many studies on acute renal injury after cardiac surgery, only a few used the machine learning method to predict AKI after cardiac surgery [20, 21, 22]. However, there are only a few studies on patients with HFpEF undergoing isolated CABG by machine learning. In this study, we used a new machine learning method, to make a risk prediction model for this group of patients to better predict the occurrence of AKI following CABG surgery to decrease morbidity and mortality in these patients.

The eGFR, EF and age are important risk factors for predicting postoperative AKI in this study, which is consistent with many previous studies [1, 2, 16]. The eGFR is an index to reflect the basic function of the kidney. An abnormal eGFR prior to surgery indicates poor renal function and a group of patients who will be more prone to acute kidney injury after surgery. Increased age is a risk factor for AKI as the renal function of the human body gradually declines with age. EF is an important indicator of cardiac function, and a low EF leads to low renal perfusion, which can lead to oliguria and is more prone to acute renal injury [19, 23, 24, 25].

Although LDH is not specifically produced by kidney, it can predict the occurrence of renal injury, as noted in previous studies [22]. Previous studies have not found that preoperative albumin is a risk factor for predicting AKI after cardiac surgery. However, previous studies [26] have suggested that albumin infusion before CABG can reduce the occurrence of acute renal injury after surgery. In addition, studies [27] have shown that the increase of albumin absorption by renal tubules can reduce the occurrence of AKI. Albumin, a risk factor found in our study, can be used to predict AKI after bypass surgery. It may further improve the prediction of acute kidney injury and identify patients with potential risks at an early stage. In addition, our results also show that uric acid is an independent risk factor for AKI. Tang H *et al*. [28] showed that when uric acid is increased before cardiac surgery, there is an increased risk of AKI after cardiac surgery. Previous studies have also found that increased preoperative uric acid levels is an independent risk factor for AKI after cardiac surgery [29].

There are limitations of this study. Our study was a single-center, retrospective study, with some selection bias. However, all the included patients were HFpEF, and were not compared with other heart failure patients. In the future, we will try to increase these variables to further improve the prediction model. In this study, the diagnosis of AKI was based on KDIGO criteria. Since diuretics are used in many patients after surgery, urine volume was not used as one of the diagnostic criteria of AKI.

## 5. Conclusions

Ejection fraction, estimated glomerular filtration rate, age, albumin, uric acid, and lactate dehydrogenase are independent risk factors for acute kidney injury in heart failure preserved ejection fraction patients after coronary artery bypass grafting by the random forest model. The application of machine learning can better predict clinical events.

## Data Availability

The datasets used and/or analyzed during the current study are available from the corresponding author on reasonable request.

## Abbreviations

CABG, coronary artery bypass grafting; AKI, acute kidney injury; KDIGO, Kidney Disease Improving Global Outcomes; ROC, receiver operating characteristic; eGFR, estimated glomerular filtration rate; IABP, intra-aortic balloon pump; CI, confidence interval; ICU, intensive care unit; BMI, body mass index; PCI, percutaneous coronary intervention; LVEF, left ventricular ejection fraction; IQR, interquartile range; OR, odds ratio.
